# Adult Case of Pontocerebellar Hypoplasia without the Claustrum

**DOI:** 10.3390/neurolint16050085

**Published:** 2024-10-07

**Authors:** Koji Hayashi, Shiho Mitsuhashi, Ei Kawahara, Asuka Suzuki, Yuka Nakaya, Mamiko Sato, Yasutaka Kobayashi

**Affiliations:** 1 Department of Rehabilitation Medicine, Fukui General Hospital, 55-16-1 Egami, Fukui 910-8561, Japanyouyou1996ha@gmail.com (Y.N.); satomoko@f-gh.jp (M.S.); 2 Department of Pathology, Fukui General Hospital, 55-16-1 Egami, Fukui 910-8561, Japan; ei@kawahara-fam.jp; 3 Graduate School of Health Science, Fukui Health Science University, 55-13-1 Egami, Fukui 910-3190, Japan; yasutaka_k@fukui-hsu.ac.jp

**Keywords:** pontocerebellar hypoplasia, claustrum, adult

## Abstract

We describe the case of a 63-year-old man with pontocerebellar hypoplasia without the claustrum (CL). The patient had a history of cerebral palsy, intelligent disability, cerebellar atrophy, and seizures since birth. At age 61, brain computed tomography (CT) revealed significant cerebellar and brainstem atrophy. At age 63, he was admitted to our hospital for aspiration pneumonia. Although he was treated with medications, including antibiotics, he died one month after admission. The autopsy revealed a total brain weight of 815 g, with the small-sized frontal lobe, cerebellum, and pons. The cross-section of the fourth ventricle had a slit-like appearance, rather than the typical diamond shape. In addition, bilateral CLs were not observed. Apart from CL, no other missing brain tissue or cells could be identified. Microscopic examinations disclosed neurofibrillary tangles in the hippocampus but not in the cortex; however, neither senile plaques nor Lewy bodies were detected. No acquired lesions, including cerebral infarction, hemorrhage, or necrosis, were noted. We pathologically diagnosed the patient with pontocerebellar hypoplasia without CL. As there have been no prior reports of pontocerebellar hypoplasia lacking CL in adults, this case may represent a new subtype. Congenital CL deficiency is likely associated with abnormalities in brain development. CL may play a role in seizure activity, and the loss of bilateral CLs does not necessarily result in immediate death. Further studies are needed to clarify the functions of CL.

## 1. Introduction

The claustrum (CL) is a thin layer of neurons and glial cells that connects with the cerebral cortex and various subcortical regions, including the amygdala, hippocampus, and thalamus [1,2]. It is situated between the insular cortex on the lateral side and the putamen on the medial side, and is enclosed by the extreme and external capsules, respectively [2]. CL is found in all mammalian species, from insectivores to humans, although its exact shape and some of its connections seem to vary across different species [3]. It is a very small tissue—in humans, its volume is only 0.4 percent of the cerebral cortex—but it is interconnected with many different areas of the cortex [3,4]. It has been reported that CL has reciprocal connections with both allo- and neocortical regions, including the frontal, premotor, ventral anterior cingulate, hippocampus, and entorhinal cortex, as well as the temporal, occipital, sensory, and motor areas [4]. Additionally, it connects with subcortical structures such as the thalamus, basal ganglia, caudate nucleus, putamen, globus pallidus, and lateral amygdala [4]. It is evident that CL sits at the intersection of numerous simple loops with the cortex [5]. The function of CL is not fully elucidated, but it has been reported that CL may related to schizophrenia [6], epilepsy [4,7], consciousness [8,9], parkinsonism [10], and stress/anxiety [11]. In this report, we describe a case of pontocerebellar hypoplasia with congenital CL deficiency who survived into his sixth decade.

## 2. Case Presentation

This study was conducted in accordance with the Declaration of Helsinki. The patient’s family provided informed consent for all study procedures, post-mortem pathological examinations, and publication of this case report. This study was approved by the Ethical Review Committee of Nittazuka Medical Welfare Center (approval no. 2024-34).

**Figure 1:**
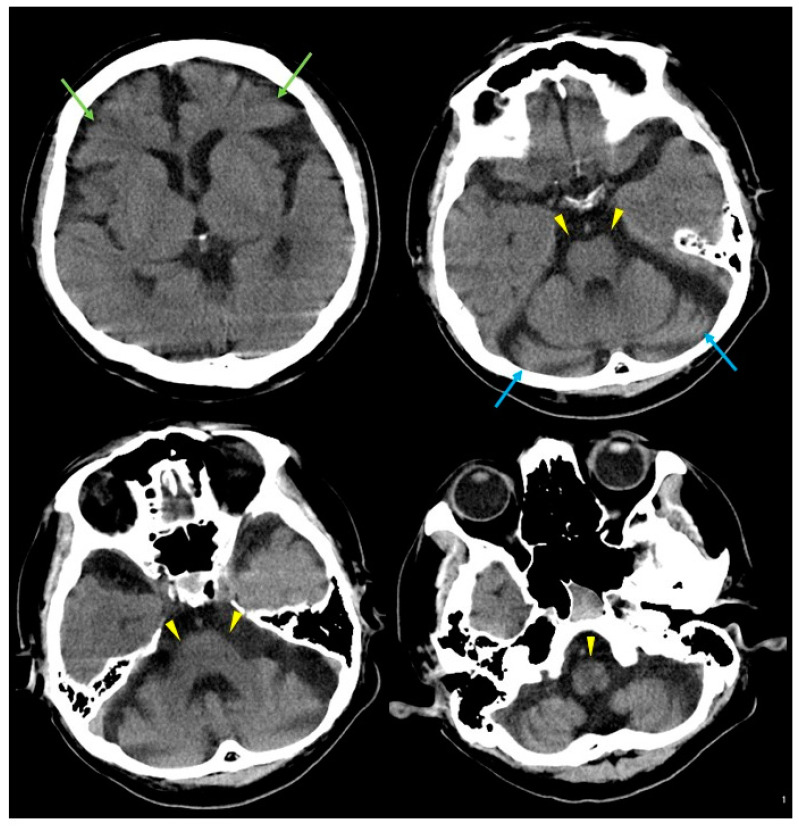
Brain computed tomography (CT) results at age 61. Brain CT showing mild frontal lobe atrophy (green arrows) and marked atrophy of the cerebellum (blue arrows) and brainstem (yellow arrowheads).

**Figure 2:**
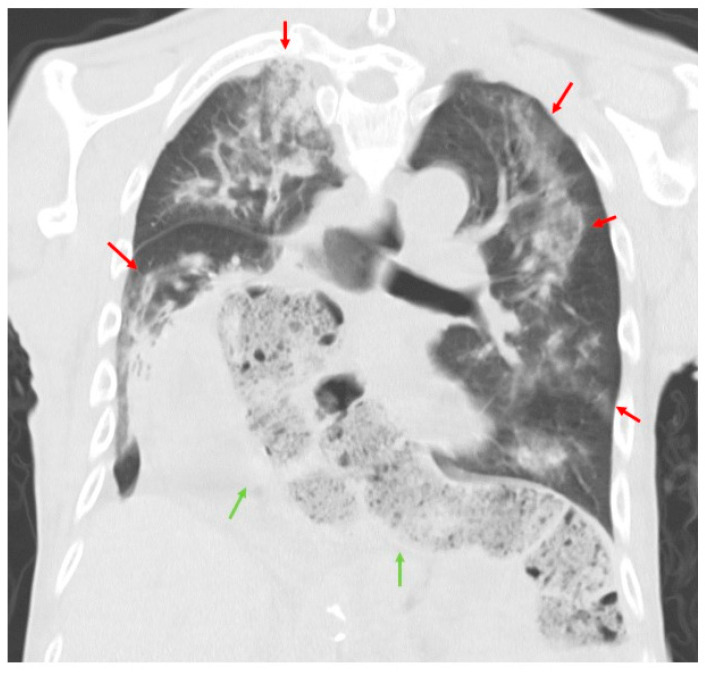
Chest CT results on admission. Chest CT showing diffuse infiltrates in both lungs (red arrows) and a severe sliding hiatal hernia (green arrows).

**Figure 3:**
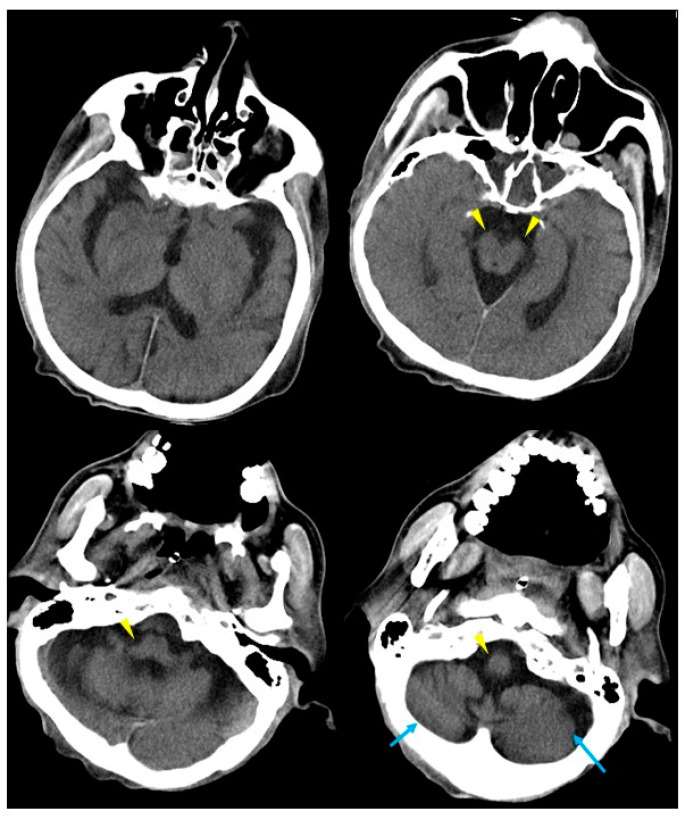
Autopsy imaging from brain CT at age 63. Autopsy brain CT showing marked atrophy of the cerebellum (blue arrows) and brainstem (yellow arrowheads), with no significant changes compared to the CT results from two years earlier.

**Figure 4:**
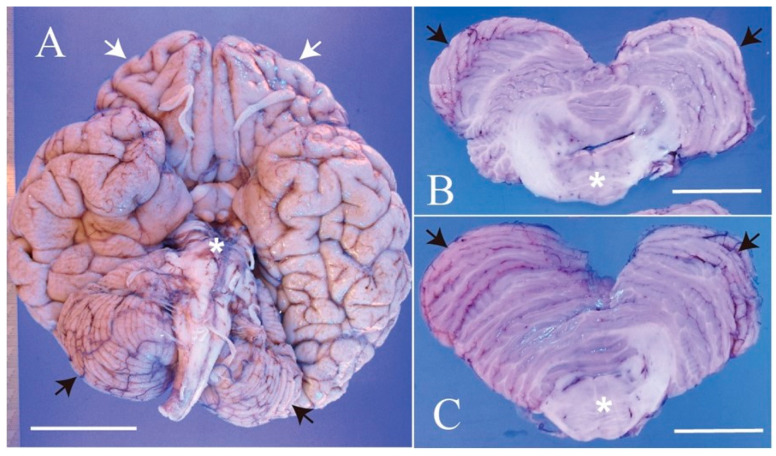
Autopsy findings of the brain. (**A**) Macroscopically, the whole brain appears reduced in size, with particularly noticeable hypoplasia in the frontal lobe (white arrows), cerebellum (black arrows), and pons (asterisk). (**B**,**C**) Cut surfaces of the cerebellum and pons. The cerebellar hemispheres (white arrows) and pons (asterisk) show hypoplasia. The shape of the 4th ventricle between the cerebellum and pons is abnormal. Scale: 5 cm.

**Figure 5:**
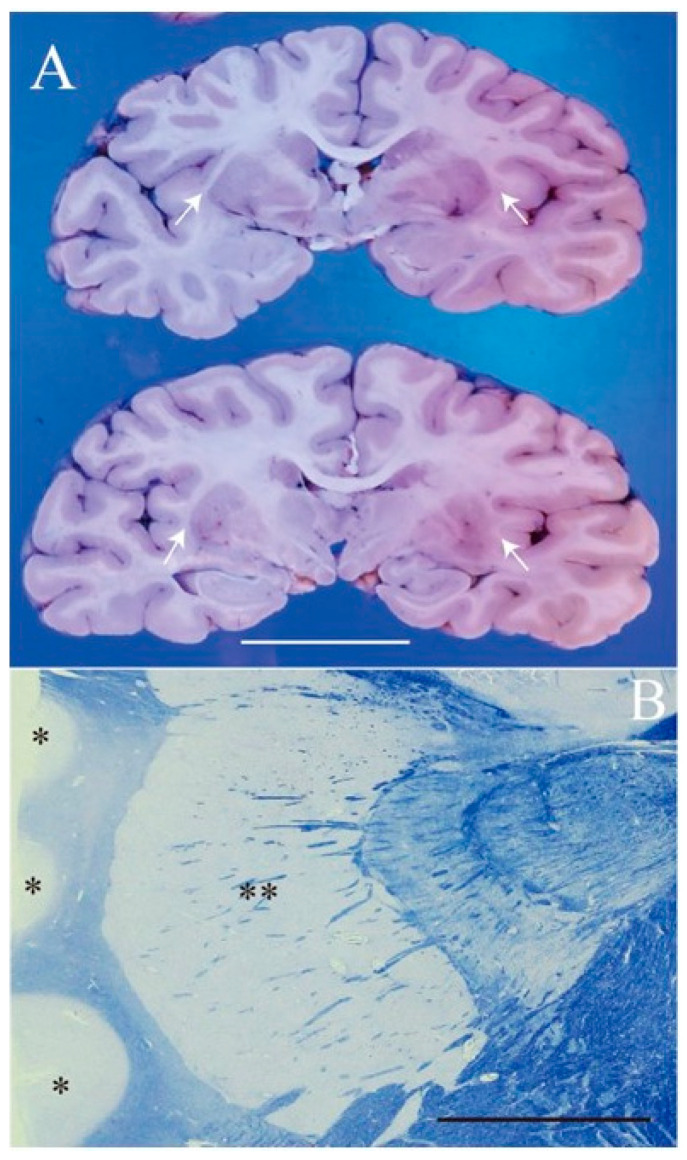
Claustrum aplasia. (**A**) Coronary sections of the cerebrum at the plain through mammillary body (lower) and the plane 7 mm forward (top). Both planes show complete aplasia of bilateral claustrum in the fused external and extreme capsules (white arrows). (**B**) Klüver–Barrera staining of the thin section shows no gray matter between cortices of the insula (asterisk) and putamen (double asterisk). White scale: 5 cm. Black scale: 1 cm.

**Figure 6:**
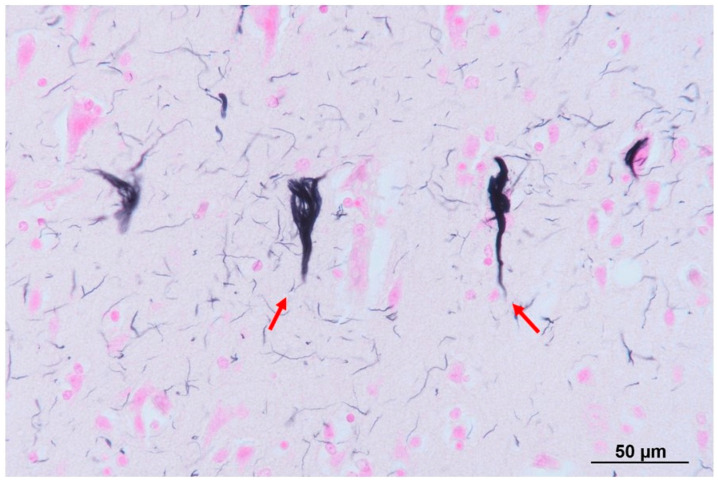
Microscopic findings in the hippocampus with Gallyas–Braak stain. A few neurofibrillary tangles (NFTs) were noted in the hippocampus (red arrows). NFTs were not observed in the cortex.

The patient was a 61-year-old man, who was a long-term resident of a facility for disabled individuals, who visited our hospital for swallowing evaluation. His medical history included cerebral palsy, intelligent disability, cerebellar atrophy, and seizures since birth, as well as gastroesophageal reflux disease and a hiatal hernia diagnosed at age 51. However, detailed medical records were unavailable, including information regarding the etiology of his cerebral palsy and seizures. He was treated with a total of nine medications, including valproic acid (800 mg/day), levetiracetam (2000 mg/day), phenytoin (200 mg/day), levocarnitine, intestinal regulators, antacids, laxatives, and expectorants. Neurological examinations revealed that the patient was conscious but frequently shouted incoherently and produced groaning sounds, making it difficult to follow instructions. There were no restrictions in eye movement, but mouth opening was limited to a width of 2.5 finger-breadths. The swallowing reflex was impaired, and although tongue muscle strength was unknown, spontaneous tongue movement was observed. The patient had severe limb joint contractures, an athetoid posture in the left hand, spasticity and rigidity in all four extremities, and was able to stand and walk with assistance. Brain computed tomography (CT) revealed moderate atrophy in the cerebellum and brainstem, while the cerebral hemispheres were relatively spared except for the frontal lobes (Figure 1). He was assessed as being able to tolerate oral intake with a gelatin diet based on a fiberoptic endoscopic evaluation and a videofluoroscopic examination of swallowing. At age 63, he developed a fever and nausea and was transported to our hospital. On admission, his vital signs were recorded as a body temperature of 39.1 °C, blood pressure of 74/44 mmHg, pulse rate of 72 beats per minute, and transcutaneous oxygen saturation (SpO_2_) of 86% on room air. Blood tests revealed an elevated white blood cell count of 12,600/µL (reference range: 3300–8600), a neutrophil percentage of 90.2% (reference range: 40.0–70.0), and a C-reactive protein level of 2.68 mg/dL. Chest CT showed bilateral pneumonia in addition to a severe sliding hiatal hernia (Figure 2). He was diagnosed with severe pneumonia and treated with antibiotics (ampicillin/sulbactam 9 g/day). Despite receiving appropriate medical treatment, he died on day 30 after admission. Postmortem brain CT imaging revealed significant cerebellar and brainstem atrophy, while the cerebral hemispheres were relatively spared (Figure 3). His family consented to an autopsy, which was performed immediately after his death. The autopsy revealed a total brain weight of 815 g, with atrophy or hypoplasia in the frontal lobe, cerebellum, and pons (Figure 4). The cross-section of the fourth ventricle did not display the characteristic diamond shape but instead had a slit-like appearance. Microscopically, no necrosis or degeneration was observed in the cerebellum and pons. The small size of the cerebellum and pons suggested congenital pontocerebellar hypoplasia. Additionally, the bilateral CLs were completely absent (Figure 5). Apart from the CL, no other missing brain tissue or cells could be identified. Moreover, microscopic examination showed neurofibrillary tangles in the hippocampus but not in the cortex (Figure 6). Neither senile plaques nor Lewy bodies were detected. No acquired lesions, including cerebral infarction, hemorrhage, or necrosis, were noted. We pathologically diagnosed the patient with pontocerebellar hypoplasia without CL. We were unable to perform any genetic tests, including next-generation sequencing (NGS).

## 3. Discussion

We describe an adult case of pontocerebellar hypoplasia without CL. The patient had cerebral palsy, epilepsy, cerebellar atrophy, and intelligent disability since birth, and lived to the age of 63. Autopsy findings revealed CL deficiency and small-sized pons and cerebellum, but no acquired lesions including stroke or necrosis.

The development process of the CL has been elucidated in animal models [12]. During development, the group of neurons that form the CL originates deep in the brain, migrates radially outward to the surface, and then migrates inward and sits in the normal position [12]. Additionally, it has been shown that certain genetic abnormalities, including *Reelin* mutations or knockout of *Apoer2*, can cause malpositioning of CL neurons [12]. Thus, certain congenital anomalies may lead to the malpositioning or absence of the CL. Pontocerebellar hypoplasia (PCH), a congenital anomaly, can lead to severe motor and cognitive deficits from birth, potentially sharing mechanistic pathways with acquired dysfunctions, such as those observed in the Guillain–Mollaret triangle, where motor coordination is impaired due to damage to the dentato-rubro-olivary pathway [13]. Additionally, cerebellar and brainstem atrophy in adults can be associated with acquired conditions, including spinocerebellar degeneration (SCD). Differentiation from SCD was necessary in this case. Histopathological analysis in SCD reveals a marked loss of neurons, predominantly Purkinje cells in the cerebellum, as well as in other regions of the central nervous system, including the pons, spinal cord, vermis, dentate nucleus, and medulla [14]. As there was no neuronal loss, including Purkinje cells, and no other acquired lesions identified in our case, the findings suggest a congenital origin. Furthermore, the absence of CL can be seen in developmental abnormalities [12], suggesting that our case is likely due to a congenital anomaly.

To the best of our knowledge, only eight autopsy cases of congenital CL deficit have been reported, and we summarized their clinicopathological findings in Table 1 [15,16,17,18,19]. The clinical diagnoses include arthrogryposis multiplex congenita [15], pontocerebellar hypoplasia [16], and lissencephaly (with muscular dystrophy) [17,18,19]. All eight previously reported cases had some brain malformations, and died at an early age, at ages between 4 days and 16 months. In all cases, the CL was completely absent, although one case had a rudimentary claustrum [15]. Many cases have muscular disorders, respiratory failure, and physical deformity. In five out of nine cases including our case, seizure developed [16,17].

Since all of these cases had some form of brain malformation, it is possible that normal CL formation was disrupted by the presence of these malformations. The cases we reviewed included lissencephaly, a condition potentially associated with abnormalities in the Reelin protein [20]. Reelin, encoded by the Reelin gene, is involved in proper CL positioning [12]. CL deficiency has been reported as a common finding in lissencephaly [17,21]. In contrast, CL deficiency is unusual in pontocerebellar hypoplasia, which is a unique feature in our case [16]. Only one report has described the absence of the CL in pontocerebellar hypoplasia, but in that case, two siblings died within a few days [16]. Because there have been no reports of adult cases of pontocerebellar hypoplasia without CL, our case appears to represent a new subtype of pontocerebellar hypoplasia. Although the previous report, like our case, did not include genetic testing, the authors found that *Pax*6*sey*/*Pax*6*sey* mutant mice can develop developmental anomalies involving both cerebellar and cerebral malformations [16,22,23]. While it cannot be determined with certainty that this gene is the cause, it is possible that a genetic background distinct from lissencephaly may have contributed to the development of pontocerebellar hypoplasia lacking the CL.

Epilepsy is also believed to be linked to the CL [4,7], and many studies for its potential role in seizures and awareness have been conducted previously. Although its precise functions remain unclear, the CL is hypothesized to synchronize electrical activity across different brain regions, which may contribute to consciousness [2,4,24]. Studies using electroencephalogram (EEG) and functional magnetic resonance imaging (fMRI) have shown increased brain activity in the CL during interictal epileptiform discharges in patients with epilepsy, suggesting a possible involvement in seizure dynamics [25,26,27,28]. Electrical stimulation of the CL in animals and humans has demonstrated altered awareness, including unresponsiveness and memory lapses, further supporting its role in seizures [29,30]. Clinical observations also show transient CL changes in patients experiencing status epilepticus and focal seizures [4]. It has been reported that several cases had reversible MRI abnormalities in the CL during acute seizures [31], indicating a potential link between CL and seizure manifestations. Furthermore, kindling models and experimental studies have implicated the CL in seizure initiation and propagation [4,32,33,34,35], with some evidence suggesting that CL involvement may contribute to impaired consciousness during seizures. Although there is evidence linking various seizures with the CL, it is unclear whether epilepsy is solely associated with CL deficiency, because congenital CL deficiency often occurs concomitantly with brain malformations.

All previously reported cases we summarized resulted in death at a young age. Apart from the cases of congenital bilateral CL deficiency, we identified three previously reported cases of acquired bilateral CL involvement. Ishii et al. reported a case of bilateral CL involvement caused by mumps virus encephalitis [36]. The patient experienced encephalitic symptoms, including fever, headache, psychological disturbances, and seizures. T2-weighted brain MRI revealed reversible hyperintensities in the bilateral CLs. The patient was successfully treated with antiepileptic drugs and major tranquilizers. Similarly, Sperner et al. reported a case of bilateral CL involvement due to non-viral encephalopathy [37]. The patient developed status epilepticus, behavioral symptoms of psychosis, and severe cognitive impairment following two weeks of headaches, dizziness, fatigue, and low-grade fevers up to 38 °C. T2-weighted brain MRI also revealed reversible hyperintensities in the bilateral CLs. The patient was treated with acyclovir, antibiotics, and dexamethasone, and all symptoms resolved. Matsuzono et al. reported a case of bilateral CL involvement due to anti-glutamic acid receptor (anti-GluR) antibody-positive encephalitis [38]. The patient presented with altered consciousness, myoclonus, and parkinsonism, following arthralgia in both distal interphalangeal joints, hearing loss and tinnitus in the left ear, and gait disturbance. T2-weighted fluid-attenuated inversion recovery (FLAIR) magnetic resonance imaging (MRI) revealed reversible hyperintensities in the bilateral CLs, medial aspects of the anterior lobe, and periventricular lesions. The patient was treated with oral prednisolone, plasma exchange therapy, and methylprednisolone pulse therapy, and was able to return home with restored consciousness and preserved cognitive function. Although bilateral CL lesions were transient in all three cases, the prognosis was good in all cases. In addition, the fact that our patient survived into his 60s suggests that bilateral CL deficiency is not associated with immediate death.

There are two major limitations in this report. The first is that genetic testing was not conducted. Although we suspected a new type of pontocerebellar hypoplasia, a genetic diagnosis was not possible. Had genetic testing, such as NGS, been performed, it could have provided more compelling insights. The second limitation is that much of the patient’s medical history and brain imaging, particularly brain MRI, was unavailable. The lack of information from birth made it difficult to fully compare this case with other reported cases. After examining the pathological findings, we suspected that the patient had a congenital CL deficiency, but we were unable to review previous images. If this information had been more comprehensive, the presentation would have been even more compelling.

## 4. Conclusions

As there have been no prior reports of pontocerebellar hypoplasia lacking CL in adults, this case may represent a new subtype. Based on previous reports, congenital CL deficiency is likely associated with abnormalities in brain development. The CL may play a role in seizure activity. While earlier cases of pontocerebellar hypoplasia with CL deficiency have shown that patients typically died shortly after birth, this patient survived to an advanced age, indicating that the loss of the bilateral CLs does not necessarily result in immediate death. This report may contribute new insights into the function of the CL and the effects of CL deficiency. Further studies are needed to clarify the functions of the CL.

## Figures and Tables

**Table 1:** Summary of the clinicopathological features of cases without CL congenitally.

Patient (No.)	Ref.	Gender (M/F)	Premature Birth (Y/N)	Symptoms by Nature	Malformation by Nature	Clinical Diagnosis	The Cause of Death	Age of Death	Brain Malformation	CL
1	[15]	F	N	cyanosis and abnormal respiration	micrognathos, bilateral equino-varus deformities of the feet, whilst the hands were flexed and held in ulnar deviation	arthrogryposis multiplex congenita	purulent bronchitis and bronchopneumonia	16 months	cerebral malformation	Absent, but a claustral rudiment lay was noted
2	[16]	F	N	respiratory failure, no suction reflex, increasing generalized hypertonia and seizures	severe reduction in the cerebral volume and much widened subarachnoidal spaces	pontocerebellar hypoplasia	status epilepticus led to intractable desaturation	5 days	small neocerebellar hemispheres, volumetric reduction in the cerebral cortex and white matter (pallium), thickened the meninges, small and simplified cortical convolutions	Absent
3	[16]	F	N	no suction reflex, difficulty of oral feeding, tonic seizures, apnea, bradycardia and O2-desaturation, exaggerated startle response (hyperacousis)	severe reduction in the cerebral volume and much widened subarachnoidal spaces	pontocerebellar hypoplasia	convulsions	22 days	small neocerebellar hemispheres, volumetric reduction in the cerebral cortex and white matter (pallium), thickened the meninges, small and simplified cortical convolutions	Absent
4	[17]	F	N.D.	seizure and hypertonia of limbs	N.D.	lissencephaly, type I	infection	11.5 months	microcephalic and agyria	Absent
5	[17]	F	N	growth deficiency, severe psychomotor retardation followed by seizure	microcephaly and facial dysmorphism	Miller–Dicker syndrome, lissencephaly, type I	acute biventricular heart failure	9 months	microcephalic and agyria	Absent
6	[17,18]	F	N	severe muscular hypotonia	N.D.	muscular dystrophy, lissencephaly, type II	cerebral dysregulation	9 months	agyria, polymicrogyria, pachygyria, absent of cerebral peduncles, Dandy–Walker malformation	Absent
7	[17,19]	M	N	severe asphyxia, muscular hypotonia, poor sucking	N.D.	muscular dystrophy, lissencephaly, type II	bronchopneumonia	14 months	argyria, polymicrogyria, pachygyria, absence of olfactory nerve, cerebral peduncles and inferior vermis, hypoplasia of the superior cerebellar vermis, Dandy–Walker malformation	Absent
8	[17]	F		asphyxia and hydrocephalus, with little spontaneous motility and poor reaction to painful stimuli	N.D.	lissencephaly, type II	cerebral dysregulation	4 days	agyria, polymicrogyria, pachygyria, absent of cerebral peduncles, Dandy–Walker malformation	Absent
9	This report	M	N.D.	intelligent disability and seizures	cerebellar atrophy	cerebral palsy, cerebellar atrophy	aspiration pneumonia	63 years	atrophy of the frontal lobe, cerebellum and pons	Absent

## Data Availability

The original contributions presented in the study are included in the article, further inquiries can be directed to the corresponding author.
